# Snakebite envenomation turns again into a neglected tropical disease!

**DOI:** 10.1186/s40409-017-0127-6

**Published:** 2017-08-08

**Authors:** Jean-Philippe Chippaux

**Affiliations:** 1 CERPAGE, Faculté des Sciences de la Santé, Université d’Abomey-Calavi, Cotonou, Benin; 2 UMR216, Mère et enfant face aux infections tropicales and PRES Sorbonne Paris Cité, Université Paris Descartes, Faculté de Pharmacie, Paris, France; 3 Institut de Recherche pour le Développement, Cotonou, Benin

## Abstract

On June 9^th^, 2017 WHO categorized snakebite envenomation into the Category A of the Neglected Tropical Diseases. This new situation will allow access to new funding, paving the way for wider and deeper researches. It should also expand the accessibility of antivenoms. Let us hope that it also leads to cooperation among stakeholders, aiming at improving the management of snakebites in developing countries.

Removed from the Neglected Tropical Diseases (NTD) list in 2013 [1], recognition by the World Health Organization (WHO) of snakebites in the category A of the Neglected Tropical Diseases finally became a reality on June 9^th^, 2017. This has been requested for many years by all stakeholders dealing with this world scourge that particularly affects developing countries. Snakebite is one of the most important NTDs in terms of both incidence and severity, and its clinical characteristics have readily served as a basis for advocacy. Of course, it also occurs in industrialized countries and even outside the tropics [2, 3], but more than 95% of cases do take place in tropical and/or developing countries.

Snakebites disproportionately involve the poorest of the poor, mostly in rural areas. Although very expensive, provision of treatment for the majority of cases does not bring sustainable profit to manufacturers – and this is the main reason for the current antivenom shortage [4]. The African Society of Venimology (ASV) has proposed a strategy to alleviate the shortage that addresses four main challenges [5].

The first challenge, now, is to specify the requirements for antivenom at an operational, local level. In order to do this, it is essential to organize the collection of epidemiological data, which will enable seasonal anticipation of snakebite number and location [6]. The only continent where this objective can be considered achieved is America [7]. In Asia, some data exist in India, for example [8], but they do not suffice to bring a consistent view of the situation. Analyses of African data have indicated that the problem there is of much underestimated magnitude [9], and this is largely being confirmed by the first epidemiological analyses from national health registries [10].

The second challenge is education of the at-risk population. In Africa especially, as in many parts of Asia, most snakebite victims go to traditional healers, rather than to health centers, to receive treatment. This happens because the cost of medical care is out of proportion to the average income of a family of farmers. The result of this situation is inappropriate delay in administration of antivenom, which ultimately increases the rate of complications and the cost to society.

The third challenge is to improve the accessibility of antivenoms. An antivenom is a complex biological product that is neither a drug nor a vaccine [11]. It cannot be a generic: the antibodies that compose it are produced by an animal (most often a horse) after it has been immunized with the appropriate venoms, i.e., those from the region where the antivenom will be used. The antibodies should be purified by enzymatic and possibly physicochemical processes. These processes, as well as lyophilization and bottling, should ideally take place in a manufacturing setting that adheres to good manufacturing practices. In addition, antivenom should be validated by a clinical study in humans before it can be marketed [11]. Taken together, these steps are very costly, but compromise can be dangerous. In Africa for instance, antivenom must be effective against all regional snakes, safe enough to use in isolated health centers, stable in the heat (lyophilized), affordable, and accessible when and where it is needed. If the development cost cannot be reduced, then all stakeholders (national and local governments, private enterprises, medical educators and providers, etc.) must work together to improve accessibility and reduce the per-patient price.

The fourth and final challenge is training of health personnel, including physicians, nurses, and public health professionals. Selection, distribution and use of appropriate antivenoms by local professionals are essential to resolve the current crisis.

It is undeniable that the recognition of snakebite by WHO as a NTD-A results from the role and intervention of many. The most visible advocates for this change have included international NGOs such as Médecins Sans Frontières (MSF), Health Action International (HAI), and the Global Snakebite Initiative (GSI), through their networks, reputation and resources. However, great credit is also due to the numerous doctors, scientists, and health authorities who have kept the field alive for decades in the absence of proper recognition, and to grassroots organizations such as ASV, which have alerted local governments, manufacturers and healthcare systems to the need for improvement.

The WHO, governments worldwide, and humanitarian foundations will now have an incentive to provide resources to NGOs, academics, and grassroots organizations to pursue the shared goal of reducing suffering and death attributable to the longstanding antivenom shortage. Complementary work at all levels is not only possible, but essential, if we are to succeed.

## Abbreviations

ASV: African Society of Venimology
GSI: Global Snakebite Initiative
HAI: Health Action International
MSF: Médecins Sans Frontières
NGO: Non-governmental organization
NTD: Neglected tropical disease
WHO: World Health Organization
